# Three Specific Potential Epitopes That Could Be Recognized by T Cells of Convalescent COVID-19 Patients Were Identified From Spike Protein

**DOI:** 10.3389/fimmu.2022.752622

**Published:** 2022-01-28

**Authors:** Yunwen Zhang, Zhengrong Yang, Mingyuan Tang, Hao Li, Tian Tang, Guilian Li, Yifan Zhong, Xiaomin Zhang, Xiaohui Wang, Chuan Wang

**Affiliations:** ^1^ West China School of Public Health and West China Fourth Hospital, Sichuan University, Chengdu, China; ^2^ Research and Transformation Center for Poisoning Treatment, Laboratory Science & Precision Prevention and Treatment, West China-PUMC C.C. Chen Institute of Health, Sichuan University, Chengdu, China; ^3^ AIDS Prevention and Control Department, Shenzhen Center for Disease Control and Prevention, Shenzhen, China

**Keywords:** SARS-CoV-2, spike protein, cross-reaction, HLA, T-cell epitope

## Abstract

The current coronavirus disease 2019 (COVID-19) vaccines are used to prevent viral infection by inducing neutralizing antibody in the body, but according to the existing experience of severe acute respiratory syndrome coronavirus (SARS) infection, T-cell immunity could provide a longer durable protection period than antibody. The research on SARS-CoV-2-specific T-cell epitope can provide target antigen for the development and evaluation of COVID-19 vaccines, which is conducive to obtain COVID-19 vaccine that can provide long-term protection. For screening specific T-cell epitopes, a SARS-CoV-2 S protein peptide library with a peptide length of 15 amino acids was synthesized. Through flow cytometry to detect percentage of IFN-γ^+^ T cells after mixed COVID-19 convalescent patients’ peripheral blood mononuclear cell with peptide library, seven peptides (P77, P14, P24, P38, P48, P74, and P84) that can be recognized by the T cells of COVID-19 convalescent patients were found. After excluding the nonspecific cross-reactions with unexposed population, three SARS-CoV-2-specific T-cell potential epitopes (P38, P48, and P84) were finally screened with the positive reaction rates between 15.4% and 48.0% in COVID-19 convalescent patients. This study also provided the HLA allele information of peptide-positive-response COVID-19 convalescent patients, thus predicting the population coverage of these three potential epitopes. Some HLA alleles showed higher frequency of occurrence in COVID-19 patients than in total Chinese population but no HLA alleles related to the T-cell peptide response and the severity of COVID-19. This research provides three potential T-cell epitopes that are helpful for the design and efficacy evaluation of COVID-19 vaccines. The HLA information provided by this research supplies reference significance for subsequent research such as finding the relation of HLA genotype with disease susceptibility.

## Introduction

Coronavirus disease 2019 (COVID-19) that is caused by severe acute respiratory syndrome coronavirus 2 (SARS-CoV-2) has led to just under 5.4 million deaths and over 278 million cases globally, as of December 26, 2021 (https://www.who.int/). SARS-CoV-2 is the seventh identified coronavirus that could cause human disease till now, and it belongs to the same genus of β-coronavirus as severe acute respiratory syndrome coronavirus (SARS-CoV) (1). Both SARS-CoV and SARS-CoV-2 cause similar clinical symptoms and disease outcome and possess similar transmission pattern. Long-term monitoring of the immune status of convalescent SARS patients revealed that their neutralizing antibody and IgG persisted for 2 years (2) but gradually declined from 2 to 3 years later until they totally disappeared (3). The memory B-cell response could not be detected after 6 years (4), but specific T-cell response remained detectable even after 17 years in some individuals (5). These results indicated that compared with antibody, memory T-cell immunity is more durable and plays a more long-term role in immune clearance of coronavirus infection. Neutralizing antibody plays an important role in antiviral immunity by blocking virus invasion, besides, T-cell-mediated immune response is also essential. CD4^+^ T cells help to induce maturation of memory B cell and antibody response (6), CD8^+^ T cells assist in virus clearance, and memory CD8^+^ T cells provide long-term protective effect and effectively prevent secondary infection (7, 8). Recently, the CD8^+^ T-cell epitopes of SARS-CoV-2 discovered by Nathan et al. showed broad protection against virus variants. Therefore, in addition to neutralizing antibody, specific T-cell response deserved more attention both during designing and evaluating the protective effect of virus vaccine. Improving the specific T-cell response induced by COVID-19 vaccines can enhance the protection of the vaccines. Monitoring the intensity and duration of specific T-cell response induced by vaccine is vital for comprehensively evaluating the protective efficacy of COVID-19 vaccine.

At present, quite a few SARS-CoV-2 T-cell epitopes have been reported, but only a part of them were confirmed by T cells of COVID-19 patients. Since the T-cell response caused by SARS-CoV-2 proteins was also detected in unexposed population (5, 9), it is necessary to detect the cross-reaction of screened epitopes in unexposed population to determine the specificity of T-cell epitopes. The spike (S) protein plays a key role in the process of virus invasion (10); therefore, this study focused on specific T-cell epitope peptides in the S protein.

In this study, we used mixed convalescent COVID-19 patients’ peripheral blood mononuclear cell (PBMC) samples to screen epitopes from an S protein overlap peptide library that could be recognized by T cells of convalescent COVID-19 patients. After excluding the nonspecific cross-reactions with unexposed population, three peptides (P38, P48, and P84) that could be specifically recognized by T cells of convalescent COVID-19 patients were screened out. Then, the recognition of these three peptides by T cells of convalescent COVID-19 patients was verified by more samples. We found that some HLA alleles showed higher frequency of occurrence in COVID-19 patients than in Chinese population, but no HLA alleles was related to T-cell response to peptide and the severity of COVID-19. In conclusion, the three specific T-cell potential epitope peptides in S protein enrich the SARS-CoV-2 T-cell epitope peptide database and provide the reference epitopes for follow-up study to identify the candidate epitope peptides of COVID-19 vaccine, the evaluation of COVID-19 vaccine immune effect, and the monitoring of specific memory T-cell immunity after virus infection. The higher frequency of occurrence of some HLA alleles in COVID-19 patients may suggest that these alleles are associated with disease susceptibility, but this needs to be verified in a large population sample.

## Materials and Methods

### General Information of Discharged COVID-19 Patients and Collection of Blood Samples

This study recruited discharged COVID-19 patients from the Shenzhen Third People’s Hospital of Guangdong Province from January to April 2020, including 71 convalescent COVID-19 patients and 6 close contacts of COVID-19 patients. Blood samples were collected within 2 weeks after discharge, and PBMCs and serum were separated immediately and stored in liquid nitrogen for PBMCs or −80°C for serum. All COVID-19 patients meet the discharge standard according to the Novel Coronavirus Pneumonia Diagnosis and Treatment Plan (Provisional 7th Edition) promulgated by the National Health Commission of the People’s Republic of China (11). We also collected PBMC samples from 17 unexposed individuals (female: 76.4%, 13/17, male: 23.5%, 4/17; age: 22–28 years old; uninfected with SARS-CoV-2 and unvaccinated of COVID-19 vaccine) as negative controls. The research process was conducted in strict accordance with ethical requirements and was reviewed by the Ethics Committee of the Shenzhen Centre for Disease Control and Prevention of Guangdong Province (QS2020070048).

### The Peptides Library of S Protein

The amino acid sequence of the SARS-CoV-2 S protein (NCBI access number: YP_009724390.1) was used to design and synthesize peptides. Peptides with a length of 15 amino acids were synthesized according to the amino acid sequence, with adjacent peptides overlapping by 5 amino acids. A total of 127 peptides (P1~P127, **Supplementary Table S1**) were obtained, synthesized by GenScript Biotech Corporation (Nanjing, China).

### Screening of Positive Epitope Peptides

The 127 peptides were arranged in a checkerboard list of 12 (C1–C12) × 11 (R1–R11) in order (**Supplementary Table S2**), and 23 peptide groups were obtained. Four convalescent COVID-19 patients with moderate disease or four unexposed individuals were randomly selected and their PBMCs were mixed as tested samples or negative control samples, respectively. The proportion of IFN-γ secreting cells in CD4^+^ T and CD8^+^ T cells was detected by flow cytometry after coincubating each peptide group with test samples or negative control samples, respectively. The proportion of CD4^+^ IFN-γ^+^ T or CD8^+^ IFN-γ^+^ T cells secreted after test samples coincubated with each peptide group (minus medium stimulation) > [CD4^+^ IFN-γ^+^ T or CD8^+^ IFN-γ^+^ T cells secreted after negative control samples coincubated with the corresponding peptide group (minus medium stimulation) + 3 × standard deviation] was considered a positive response (12). By testing one group by one group, the positive peptide groups were screened. According to the checkerboard list of peptide groups, the positive peptide groups were then cross-compared and the positive peptides were found (13, 14). For the detection of cross-reaction, the proportion of CD4^+^ IFN-γ^+^ T or CD8^+^ IFN-γ^+^ T cells secreted after PBMC samples of unexposed person coincubated with the peptide (minus medium stimulation) > (CD4^+^ IFN-γ^+^ T or CD8^+^ IFN-γ^+^ T cells secreted after PBMC samples of unexposed person incubated with the same volume of medium + 2 × standard deviation) was considered a cross-reaction positive response (15).

### Detection of IFN-γ Secreting T Cells

PBMCs at 1 × 10^6^ coincubated with each peptide group (the final concentration of each peptide was 2.5 μg/ml) or the same volume of medium (unstimulated group) for 20 h (37°C, 5% CO_2_). Five hours before the end of incubation, Golgistop™ Protein Transport Inhibitor (444724, BD, Franklin Lakes, NJ, USA) was added. Phorbol-12-myristate-13-acetate (PMA)/ionomycin as positive control incubated with PBMCs and Golgistop™ Protein Transport Inhibitor were added to coincubate for 5 h (37°C, 5% CO_2_) (PAM: 50 ng/ml, P1585, Sigma, St. Louis, MO, USA; ionomycin: 500 ng/ml, 407950, Sigma). After the coincubation, cell surface markers and intracellular cytokines were stained according to the reagent instructions (Live/Dead™ Fixable Violet Dead Cell Stain Kit, L34964, Invitrogen, Waltham, MA, USA; APC Mouse Anti-Human CD3, Clone SK7, 340440, BD; PerCP-Cy™ 5.5 Mouse Anti-Human CD4, Clone SK3, BD: 341654; PE Mouse Anti-Human CD69, Clone L78, 341652, BD; FITC Mouse Anti-Human IFN-γ, Clone 25723.11, 340449, BD; Cytofix/Cytoperm™ Fixation/Permeablization Kit, 554714, BD) and were detected by flow cytometry (BD FACSCanto™ II). A total of 150,000–200,000 lymphocytes were collected from each sample, and the data were analyzed using the software Flowjo 10.

### Identification of HLA Alleles

Genomic DNA was extracted (QIAAMP DNA Blood Mini Kit, 51104, Qiagen, Hilden, Germany) from 200 μl whole blood. HLA alleles were identified by PCR-SBT (polymerase chain reaction sequence-based typing, MJ Research PTC-225, ABI 3730XL, CapitalBio Corporation, Beijing, China).

### Determination of RBD Total Antibody

Chemiluminescence microparticle immunoassay (CMIA) technology (Caris 200 Automatic Chemiluminescence Instrument, Beijing Wantai Biotech, China) was used to detect cutoff index (COI) values (sample detection value/cutoff value) for total antibody levels against SARS-CoV-2 S-RBD in serum samples.

### Sequence Conservation Analysis

Amino acid sequences of six coronavirus spike proteins were obtained from NCBI (229E: BAL45639.1, NL63: AFD98834.1, OC43: AAA03055.1, HKU1: BBA20983.1, SARS: ABA02260.1, MERS: YP_009047204.1). The Epitope Conservancy Analysis tool of the IEDB website (http://www.iedb.org/) was used to conduct conservative analysis of the screened positive T-cell epitopes.

### HLA Population Coverage

Population coverage tool of the IEDB website was used to analyze the coverage of HLA alleles in the population.

### Data Analysis

SPSS Statistics 21, RStudio 4.1.0 and GraphPad Prism 8.0 were used for statistical analysis and plotting, and Flowjo 10 was used for flow data analysis. The Chi-square test was applied to analyze the distribution of HLA alleles in the population and the positive reaction rates of T-cell epitope peptides in the tested PBMC samples. *t*-test, Mann-Whitney *U* test, and Kruskal-Wallis *H* test were used to compare the proportion of IFN-γ^+^ T cells induced by each epitope peptide. Logistic and multinomial logistic regression models were respectively used to analyze the correlation between HLA alleles with T-cell responses to epitope peptide and clinical conditions, and the age, sex, and RBD total antibody levels were included for adjustment. When the *p*-value was less than 0.05, the difference was considered to be statistically significant.

## Result

### General Information of Convalescent COVID-19 Patients

In this study, 71 PBMC samples from convalescent COVID-19 patients were collected, of which 53 samples were tested for HLA alleles, 49 samples were tested for T-cell response to peptides, and 33 samples were tested for both. PBMC samples of 6 close contacts and 17 unexposed individuals were only tested the T-cell response to peptides. The main clinical classification of 71 convalescent patients was moderate. In order to facilitate subsequent data analysis, one critical patient was included in the severe group. We also determined the RBD total antibody levels in their serum samples. The general information of COVID-19 convalescent patients is shown in **Supplementary Table S3**.

### Screening of Specific T-Cell Epitope Peptides in S Protein

**Figure 1:**
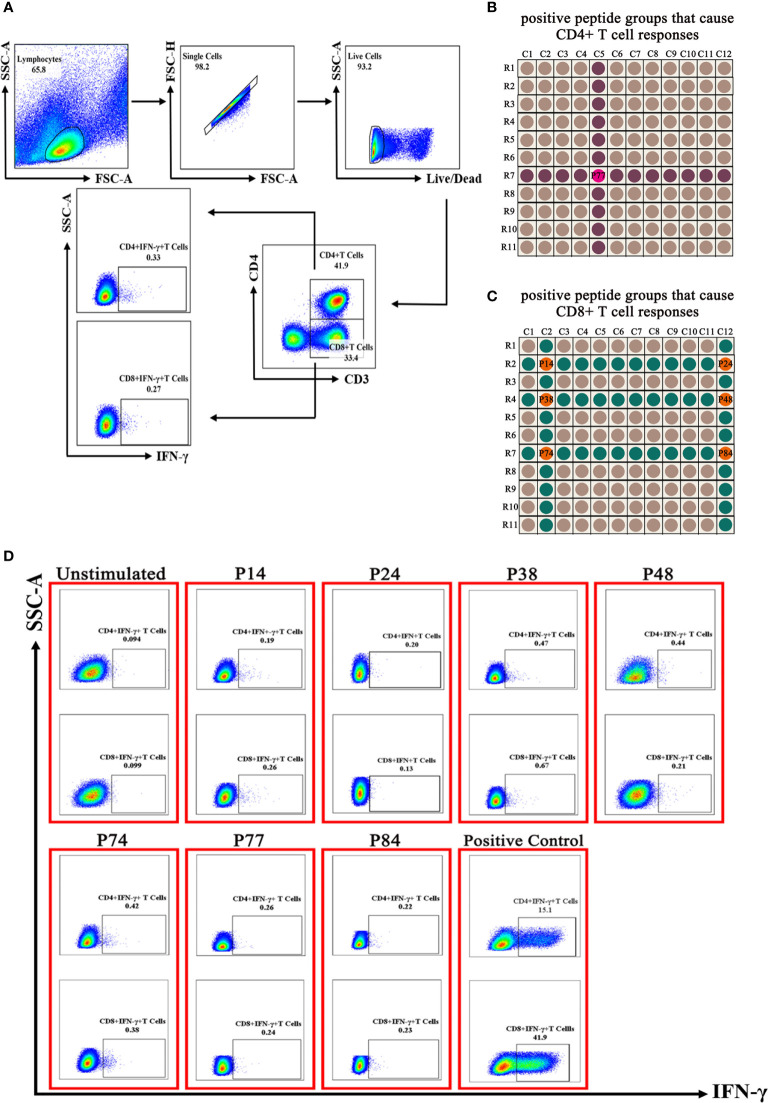
Screening results of T-cell epitope peptides by flow cytometry. **(A)** Flow gate strategy. **(B**, **C)** The epitope peptides that induced CD4^+^ IFN-γ^+^ T-cell and CD8^+^ IFN-γ^+^ T-cell-positive response were screened out by cross-comparison of the positive peptide groups. **(D)** Results showed that seven epitope peptides induced CD4^+^ IFN-γ^+^ T cell and CD8^+^ IFN-γ^+^ T-cell-positive response in PBMCs of convalescent COVID-19 patients.

In order to screen out the specific T-cell epitopes in S protein, we first synthesized a peptide library covering the S protein and divided the obtained 127 peptides into 23 peptide groups according to the chessboard list (**Supplementary Table S2**). The tested samples (randomly selected four PBMC samples of convalescent COVID-19 patients and mixed) and negative control samples (randomly selected four PBMC samples of unexposed individuals and mixed) were coincubated with each peptide group and then were detected by flow cytometry (**Figure 1A**). In the tested samples, CD4^+^ T cells showed positive response to peptide groups C5 and R7, and CD8^+^ T cells showed positive response to peptide groups C2, C12, R2, R4, and R7. Through cross-comparison in the chessboard list, positive CD4^+^ T-cell peptide P77 (**Figure 1B**) and positive CD8^+^ T cell peptides (P14, P24, P38, P48, P74, and P84) were found (**Figure 1C**), respectively. Next, we verified the seven peptides with more PBMC samples of convalescent COVID-19 patients and showed that the seven peptides could induce CD4^+^ IFN-γ^+^ T-cell and/or CD8^+^ IFN-γ^+^ T-cell-positive response (**Figure 1D**). In order to test whether the screened seven epitope peptides were specific, we tested them in PBMC samples of unexposed people. The results showed that P14 induced CD4^+^ IFN-γ^+^ T-cell and CD8^+^ IFN-γ^+^ T-cell-positive response in one PBMC sample, and P74 induced positive CD8^+^ IFN-γ^+^ T-cell response in another sample (**Supplementary Figure S1**), suggesting P14 and P74 had cross-reaction with T cells of unexposed people.

### T-Cell Responses to Seven Epitope Peptides in the Close Contacts

**Figure 2:**
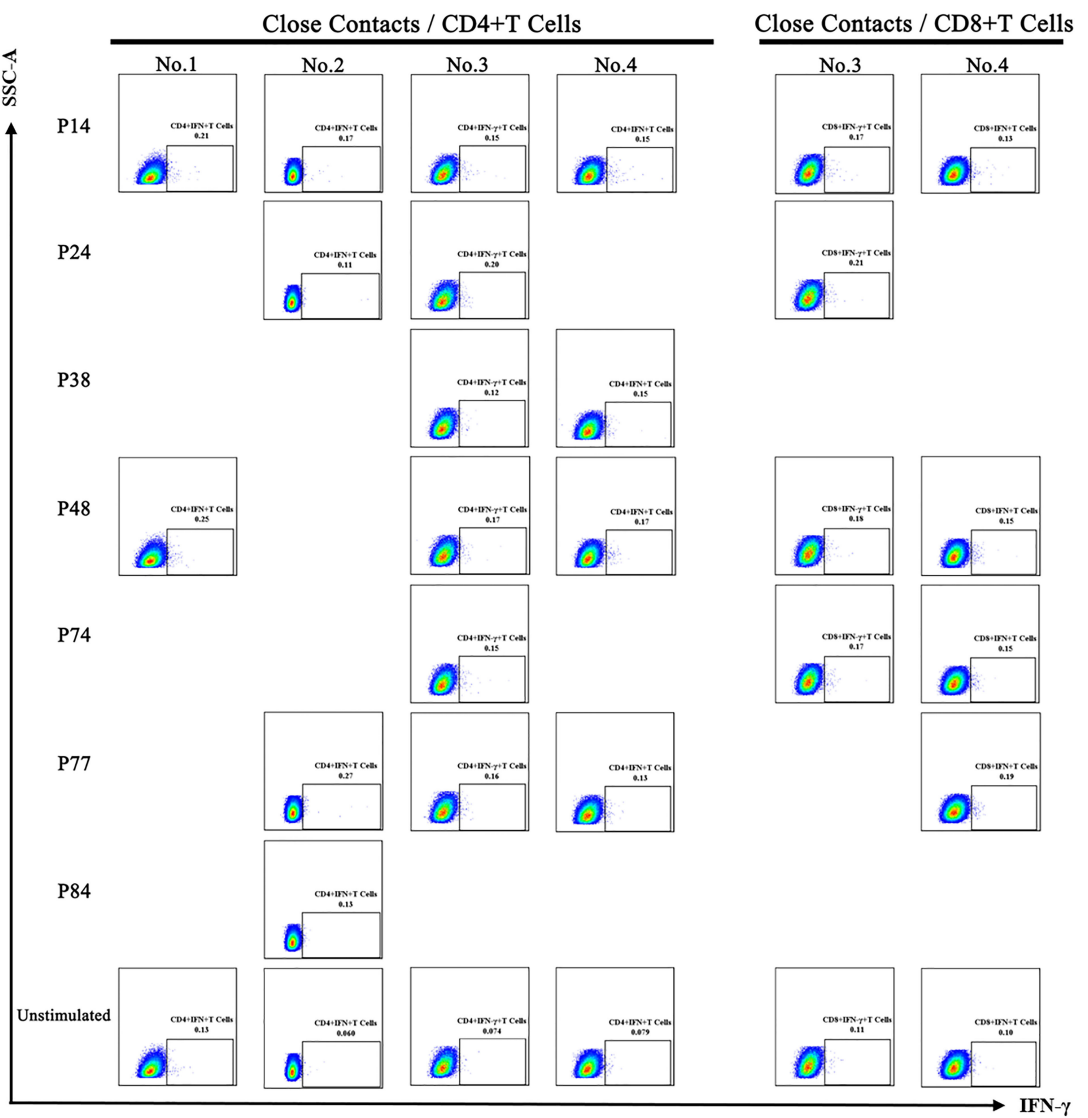
CD4^+^ IFN-γ^+^ T-cell and CD8^+^ IFN-γ^+^ T-cell responses induced by epitope peptides in close contacts. All positive T-cell response results are presented here. The seven epitope peptides (P14, P24, P38, P48, P74, P77, and P84) were tested in six PBMC samples of close contacts. The CD4^+^ IFN-γ^+^ T-cell-positive responses induced by epitope peptides were detected in four of the PBMC samples of close contacts (No. 1–No. 4). The CD8^+^ IFN-γ^+^ T-cell-positive responses induced by epitope peptides were detected in two PBMC samples of close contact human (No. 3–No. 4).

The six PBMC samples of close contacts were tested with seven screened T-cell epitope peptides individually. The results showed that CD4^+^ IFN-γ^+^ T-cell and/or CD8^+^ IFN-γ^+^ T-cell-positive response were detected in four PBMC samples (**Figure 2**). No positive T-cell response was detected in the remaining two samples.

### Sequence Characteristics of Seven Epitope Peptides

T-cell-positive responses induced by peptides P14 and P74 were detected in unexposed people; the results indicated that there was cross-reaction between these two peptides and T cells of unexposed population. The cross-reaction may be caused by the existed immune recall to some virus that they have been infected with, and the T-cell epitope peptides of ever infected virus were to certain degree homologous to the peptides screen by us. Therefore, the conservation of seven peptides were analyzed among the spike protein of four common human-infected coronavirus (229E, NL63, OC43, HKU1), SARS-CoV and MERS-CoV. The seven peptides had low conservation with common coronavirus (26.67%–46.67%) (**Supplementary Table S4**), but four of them (P38, P74, P77, and P84) had relatively high homology with SARS-CoV (≥80%) (**Table 1**). Through reviewing of the reported literature, we found P14, P24, and P77 were reported to induce T-cell response in unexposed population (9). P38 and P48 partially overlapped with the reported B-cell linear epitopes of SARS-CoV-2 (16). P38 and P74 overlapped 5 amino acids with T-cell epitopes that have been reported (17) (**Table 2**). P74 was found to induce T-cell cross-reaction in our research (**Supplementary Figure S1**). Taken together, the peptides P38, P48, and P84 screened in this study are considered potential T-cell epitopes of S protein. P14, P24, P74, and P77 were not included in the subsequent data analysis.

**Table 1:** Conservation analysis of four peptides among SARS-CoV S protein^a^.

Peptide	Peptide sequence	SARS-CoV sequence^b^	Conservation (%)
P38	SASFSTFKCYGVSPT	STFFSTFKCYGVSAT	80.00
P74	MTKTSVDCTMYICGD	MAKTSVDCNMYICGD	86.67
P77	TQLNRALTGIAVEQD	TQLNRALSGIAAEQD	86.67
P84	AGFIKQYGDCLGDIA	AGFMKQYGECLGDIN	80.00

a GenBank#: ABA02260.1.

b The red part represents different amino acids.

**Table 2:** Comparison results of peptide sequences.

Peptide	Sequence	Results of comparison	Sequence reported by literature^a^
P14	S131–145: CEFQFCNDPFLGVYY	It induced T-cell response in unexposed population (9)	CEFQFCNDPFLGVYY
P24	S231–245: IGINITRFQTLLALH	It was contained in the reported B-cell epitope (S221–245) (16)	SALEPLVDLPIGINITRFQTLLALH
		It induced T-cell response in unexposed population (9)	IGINITRFQTLLALH
P38	S371–385: SASFSTFKCYGVSPT	Partially overlapped with reported B-cell epitopes (S375–394) (16)	STFKCYGVSPTKLNDLCFTN
		Partially overlapped with reported T-cell epitopes (S381–395) (17)	GVSPTKLNDLCFTNV
P48	S471–485: EIYQAGSTPCNGVEG	Partially overlapped with reported B-cell epitopes (S480–499) (16)	CNGVEGFNCYFPLQSYGFQP
P74	S731–745: MTKTSVDCTMYICGD	Partially overlapped with reported T-cell epitopes (S721–735) (17)	SVTTEILPVSMTKTS
P77	S761–775: TQLNRALTGIAVEQD	Partially overlapped with reported T-cell epitopes (S751–765) (17)	NLLLQYGSFCTQLNR
		It induced T-cell response in unexposed population (9)	TQLNRALTGIAVEQD
P84	S831–845: AGFIKQYGDCLGDIA	Reported (18)	AGFIKQYGDCLGDIA

a The red part represents different amino acids.

### T-Cell-Positive Response to Potential Epitopes by PBMC of COVID-19 Convalescent Patients

**Figure 3:**
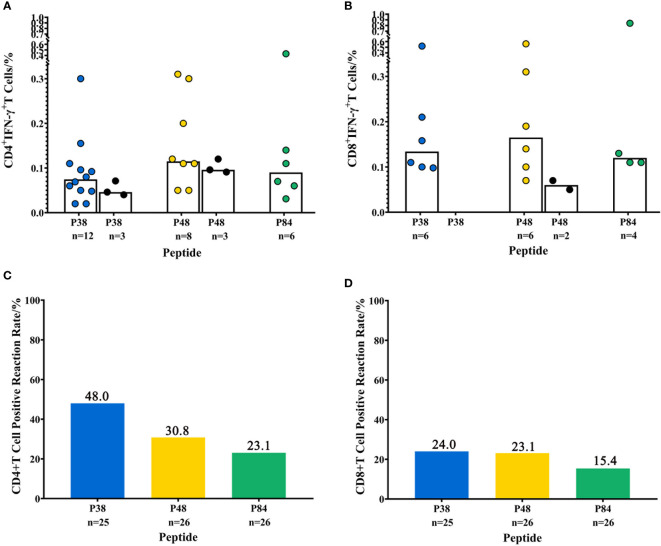
T-cell-positive response of the tested PBMC samples of convalescent COVID-19 patients to three T-cell potential epitopes (P38, P48, P84). **(A, B)** The proportion of CD4^+^ IFN-γ^+^ T cell and CD8^+^ IFN-γ^+^ T cells induced by potential epitopes (P38, P48, P84) in PBMCs of convalescent COVID-19 patients or close contacts. The columns represent the median value; the colored dots represent convalescent COVID-19 patients, and the black dots represent close contacts. The number of people who had positive T-cell responses to each epitope peptide is listed below the column. **(C, D)** The T-cell-positive reaction rates in tested PBMC samples to three potential epitopes. The number of tested PBMC samples of convalescent COVID-19 patients is listed below the column.

We validated the three potential epitopes with more PBMC samples to evaluate the level of T-cell responses induced by them. Including the positive CD8^+^ T-cell response, we also detected the positive CD4^+^ T-cell response caused by them. The results showed that the proportion value of CD4^+^ IFN-γ^+^ T cells (P38: 0.02%~0.3%, P48: 0.05%~0.31%, P84: 0.031%~0.43%) and CD8^+^ IFN-γ^+^ T cells (P38: 0.098%~0.52%, P48: 0.07%~0.56%, P84: 0.11%~0.84%) stimulated by the same peptides varied greatly among different individuals, but there was no statistically significant difference in the proportion value (median) of positive IFN-γ^+^ T cells induced by different peptides (**Figures 3A, B**). The T-cell-positive responses to P38, P48, and P84 were also detected in four close contacts (**Figure 2**). However, the proportion values of IFN-γ^+^ T cells induced by them in the close contacts were lower than those in convalescent COVID-19 patients (**Figures 3A, B**). At the same time, the proportion value of IFN-γ^+^ T cells induced by the peptides (P38, P48, and P84) in unexposed people were also significantly lower than those in convalescent COVID-19 patients (**Supplementary Figure S2**, the negative data are shown in **Supplementary Figure S3**). Due to the insufficient blood volume, only partial epitope peptides were detected in each PBMC sample of convalescent COVID-19 patients. The T-cell-positive reaction rates of convalescent COVID-19 patients to each epitope peptide are shown in **Figures 3C, D**. The results showed that there was no difference in the positive reaction rates of three epitope peptides in convalescent COVID-19 patients.

### Analysis of HLA Alleles of COVID-19 Convalescent Patients

In this study, DNA was extracted from 53 blood samples and HLA alleles were identified, including HLA-A, HLA-B, HLA-C, HLA-DRB1, HLA-DQB1, and HLA-DPB1. **Table 3** shows the HLA alleles with more than 5% of occurrence frequency in 53 COVID-19 convalescent patients. Comparing the occurrence frequency of these HLA alleles in 53 COVID-19 convalescent patients with the HLA alleles frequency database from a China Marrow Donor Program (CMDP) which included 812,211 volunteers coming from 31 provinces in China (19), it was found that among COVID-19 convalescent patients, the occurrence frequencies of HLA-A*11:01, B*46:01, B*13:01, C*01:02, C*03:04, C*06:02, DRB1*04:05, DRB1*12:01, DRB1*16:02, DQB1*03:01, DQB1*03:03, and DQB1*04:01 were higher (**Table 3**). Next, logistic regression analysis was performed on each HLA allele (frequency >5%) with T-cell responses to each potential epitope, and it was found that HLA-B*40:01 was related with the T-cell response to P48 (*p* = 0.0423); however, after adjusting for age, gender, and antibody level, the correlation was not obvious. Similarly, multinomial logistic regression analysis showed there were no alleles related to the patient’s condition. **Table 4** provides detailed HLA allele information of COVID-19 convalescent patients who had T-cell-positive responses to potential epitopes and the predicted population coverage of these alleles globally and in North Asia.

**Table 3:** The occurrence frequency of HLA alleles (frequency >5%) in 53 COVID-19 convalescent patients and in Chinese population.

Locus	HLA alleles	Frequency in COVID-19 convalescent patients (%)	Frequency in Chinese population (%)^a^	*p* ^b^
HLA-A	A*11:01	31.1	20.9	0.009
	A*24:02	16.0	15.5	0.787
	A*02:07	11.3	8.4	0.237
	A*02:01	6.6	12.0	0.102
	A*33:03	6.6	8.2	0.597
	A*30:01	5.7	6.0	0.952
HLA-B	B*46:01	16.0	10.3	0.036
	B*40:01	14.2	9.6	0.083
	B*13:01	10.4	5.0	0.008
	B*13:02	6.6	6.3	0.842
HLA-C	C*01:02	19.8	10.5	0.001
	C*03:04	17.0	6.6	0.000008
	C*07:02	11.3	10.1	0.598
	C*06:02	11.3	5.9	0.013
	C*04:01	6.6	3.8	0.119
	C*08:01	5.7	5.7	0.940
HLA-DRB1	DRB1*09:01	18.9	14.8	0.236
	DRB1*04:05	9.4	4.8	0.026
	DRB1*12:02	8.5	8.7	0.990
	DRB1*15:01	8.5	11.6	0.367
	DRB1*12:01	6.6	2.4	0.004
	DRB1*16:02	6.6	3.1	0.027
	DRB1*11:01	5.7	5.6	0.931
	DRB1*08:03	5.7	6.3	0.839
	DRB1*03:01	5.7	5.1	0.738
HLA-DQB1	DQB1*03:01	22.6	14.0	0.006
	DQB1*03:03	18.9	10.6	0.003
	DQB1*06:01	11.3	6.8	0.051
	DQB1*04:01	9.4	3.0	0.000063
	DQB1*05:02	8.5	4.9	0.070
	DQB1*02:01	5.7	3.3	0.151
HLA-DPB1^c^	DPB1*05:01	32.1	——	

a The HLA allele data come from a China Marrow Donor Program (CMDP) which included 812,211 volunteers coming from 31 provinces in China [18].

b Chi-square analysis results.

c Due to the lack of HLA-DPB1 allele distribution in Chinese population, comparative analysis was not conducted in this part.

**Table 4:** HLA allele information of convalescent patients who had T-cell-positive responses to the three potential T-cell epitopes and the predicted population coverage.

Epitope peptide	Sequence	CD4^+^ T/CD8^+^ T	Positive number/tested number	HLA alleles	HLA population coverage	
					North Asia	Globally
P38	371–385: SASFSTFKCYGVSPT	CD8^+^ T	3/17 (17.6%)	A*11:02 A*24:02 B*46:01 B*58:01 C*01:02 C*03:02	94.33%	67.99%
				A*11:01 A*24:02 B*40:01 B*51:01 C*07:02 C*14:02		
				A*02:07 A*11:01 B*46:01 B*46:01 C*01:02 C*01:02		
		CD4^+^ T	8/17 (47.7%)	DRB1*03:01 DRB1*12:02 DQB1*02:01 DQB1*03:01 DPB1*04:01 DPB1*21:01	99.47%	97.7%
				DRB1*04:05 DRB1*09:01 DQB1*03:03 DQB1*04:01 DPB1*05:01 DPB1*05:01		
				DRB1*09:01 DRB1*09:01 DQB1*03:01 DQB1*03:03 DPB1*05:01 DPB1*05:01		
				DRB1*08:03 DRB1*09:01 DQB1*03:03 DQB1*06:01 DPB1*02:02 DPB1*14:01		
				DRB1*12:01 DRB1*12:02 DQB1*03:01 DQB1*03:01 DPB1*02:01 DPB1*05:01		
				DRB1*04:08 DRB1*10:01 DQB1*03:01 DQB1*05:01 DPB1*02:01:02G DPB1*04:02:01G		
				DRB1*12:02 DRB1*16:02 DQB1*03:01 DQB1*05:02 DPB1*02:02 DPB1*107:01		
				DRB1*03:01 DRB1*09:01 DQB1*02:01 DQB1*03:03 DPB1*04:01 DPB1*05:01		
P48	471–485: EIYQAGSTPCNGVEG	CD8^+^ T	3/17 (17.6%)	A*11:02 A*24:02 B*46:01 B*58:01 C*01:02 C*03:02	96.41%	79.95%
				A*11:01 A*24:02 B*40:01 B*51:01 C*07:02 C*14:02		
				A*11:01 A*33:03 B*40:01 B*50:01 C*03:04 C*06:02		
		CD4^+^ T	3/17 (17.6%)	DRB1*03:01 DRB1*12:02 DQB1*02:01 DQB1*03:01 DPB1*04:01 DPB1*21:01	97.26%	94.64%
				DRB1*04:05 DRB1*09:01 DQB1*03:03 DQB1*04:01 DPB1*05:01 DPB1*05:01		
				DRB1*07:01 DRB1*16:02 DQB1*02:02 DQB1*05:02 DPB1*03:01:01G DPB1*14:01:01G		
P84	831–845: AGFIKQYGDCLGDIA	CD8^+^ T	4/19 (21.1%)	A*11:02 A*24:02 B*46:01 B*58:01 C*01:02 C*03:02	96.29%	80.02%
				A*02:07 A*11:01 B*15:01 B*46:01 C*01:02 C*04:01		
				A*11:01 A*24:02B*40:01 B*51:01 C*07:02 C*14:02		
				A*11:01 A*11:01 B*44:03 B*51:01 C*14:02 C*14:03		
		CD4^+^ T	6/19 (31.6%)	DRB1*03:01 DRB1*12:02 DQB1*02:01 DQB1*03:01 DPB1*04:01 DPB1*21:01	99.09%	96.59%
				DRB1*04:06 DRB1*09:01 DQB1*03:02 DQB1*03:03 DPB1*05:01 DPB1*107:01		
				DRB1*04:05 DRB1*09:01 DQB1*03:03 DQB1*04:01 DPB1*05:01 DPB1*05:01		
				DRB1*09:01 DRB1*13:02 DQB1*03:03 DQB1*06:04 DPB1*02:01 DPB1*02:02		
				DRB1*09:01 DRB1*15:02 DQB1*03:03 DQB1*05:02 DPB1*02:01 DPB1*13:01		
				DRB1*04:05 DRB1*12:01 DQB1*03:01 DQB1*04:01 DPB1*02:01 DPB1*05:01		

## Discussion

In view of the experiences acquired from convalescent SARS patients, memory B-cell responses will not provide long-term protection against viral infection over time, but memory T cell shows the advantage for long-term response. The research on SARS-CoV-2-specific T-cell epitopes has great value for the design, quality improvement, and efficacy evaluation of the COVID-19 vaccines. In this study, PBMC samples of convalescent patients of COVID-19 were used to screen T-cell epitopes of SARS-CoV-2. Seven peptides (P14, P24, P38, P48, P74, P77, and P84) that could be recognized by T cells were screened out from the S protein peptide library. Among them, four peptides were found to have a cross-reaction with unexposed people. Finally, three SARS-CoV-2-specific T-cell potential epitopes (P38, P48, and P84) were screened out.

Although the level of T-cell-positive response against SARS-CoV-2 detected in unexposed population is low (20), the presence of cross-reaction does interfere the detection of specific T-cell response. Studies have shown that the cross-reaction of epitope peptides derives from the immune recall of common coronavirus infection in unexposed people (21). Therefore, we compared the conservation of the epitope peptides screened in this study in coronavirus, and the results showed that the conservation of the screened epitope peptides was low in S protein of four common coronavirus. However, T-cell receptors can recognize the peptides that had similar biochemical and structural characteristics to induce memory T-cell response (22). In addition, cross-reaction is not only derived from similar homologous viruses but also derived from heterologous cross-reaction. For example, BCG enables the body to establish heterologous protective immunity (23). A large number of cross-reaction epitopes, including B- and T-cell epitopes, have been predicted between the diphtheria-tetanus-pertussis triple vaccine and SARS-CoV-2 (23). The ORF1ab of SARS-CoV-2 and *Mycobacterium bovis* shared similar peptides (24), and T-cell responses to ORF1ab were detected both in unexposed people and COVID-19 patients (10). However, the epidemiological research of Hensel et al. showed that BCG vaccination policy had no correlation with the spread of SARS-CoV-2 and the disease mortality (25). For the role of T-cell cross-reaction in the prognosis of COVID-19, more data are still needed to be explored.

Interestingly, four close contacts with negative nucleic acid and antibody test had positive T-cell responses after stimulation with seven peptides. Similarly, during the MERS epidemic, virus-specific T-cell responses were detected in mild patients who tested negative for antibody (26). In addition to S protein, other studies have reported that T-cell responses to other proteins of SARS-CoV-2 were detected in close contacts (27). Close contacts were exposed to the virus at a low amount level or for a short exposure time; this did not cause virus infection but caused a weak T-cell response to SARS-CoV-2.

T-cell receptor relies on HLA molecules to recognize different antigenic peptides and activate the downstream immune response, and the HLA alleles are related to the individual’s immune response. Genetic polymorphisms of HLA molecules lead to great diversity of antigen recognition and presentation in the population (28). Some HLA alleles are associated with susceptibility of different diseases (29, 30). In the research on SARS, it was found that HLA-B*46:01 and HLA-DRB1*12:02 were related to the development of SARS (31, 32), and HLA-Cw*08:01 was a susceptibility gene for SARS-CoV infection but not necessarily related to the severity of the disease (33). HLA-Cw*15:02 and DR*03:01 were protective alleles for SARS infection (34). In COVID-19, Wang et al. found the SARS-related HLA alleles did not indicate the same correlation to COVID-19 (35). In this study, the occurrence frequency of HLA-A*11:01, B*46:01, B*13:01, C*01:02, C*03:04, C*06:02, DRB1*04:05, DRB1*12:01, DRB1*16:02, DQB1*03:01, DQB1*03:03, and DQB1*04:01 in COVID-19 patients were higher than in Chinese population (19), but its significance needs to be verified in a larger sample. We also used logistic regression model to analyze the correlation of HLA alleles with the positive T-cell responses to peptides and with the disease severity. Considering that age, gender, and antibody level were correlated with immune responses of COVID-19 patients (36) and disease severity (37–39), we adjusted the model with the above-mentioned impact factors. After adjusting with these impact factors, no any allele was related with epitope peptides response or disease severity. Whether HLA alleles are related to the severity of COVID-19 are still worthy to further research, and large sample research are needed. We provided the HLA allele distribution in COVID-19 patients and the detailed HLA allele information of individuals who responded positively to the three potential specific T-cell epitopes. Although the sample size is small, these data are still helpful to further explore the effect of HLA gene polymorphism on COVID-19 susceptibility.

With the development of different types of COVID-19 vaccines, in addition to focusing on the role of neutralizing antibody, in view of the advantages of the persistence of T-cell response, induction of T-cell immunity should be an element that needs to be considered in the design for new vaccines. Screening the specific T-cell epitope peptides is an important aspect of the research on SARS-CoV-2. This study screened three potential specific T-cell epitopes and analyzed the HLA alleles of individuals with T-cell-positive responses to them. We recognize that there still exist some limitations in this study; on the one hand, it still needs more tests for confirming the accurate peptide sequences. Also, the affinity of T cell and the ability of TCR binding with epitope peptides need to be tested in the successive research, so as to further clarify the function of each epitope peptide. On the other hand, testing the common T-cell response induced by all identified epitopes among vast number of unexposed individuals to confirm the new peptide vaccine candidates is very important. However, the three T-cell potential epitopes are still helpful for the design and efficacy evaluation of COVID-19 vaccines.

## Data Availability Statement

The original contributions presented in the study are included in the article/**Supplementary Material**. Further inquiries can be directed to the corresponding authors.

## Author Contributions

CW and XW conceived and designed the research. YuZ, ZY, MT, HL, TT, GL, YiZ, and XZ performed the experiments and acquired, interpreted, and analyzed the data. YuZ, CW, and XW wrote the manuscript. All the authors read and critically reviewed the manuscript. All authors listed have made a substantial, direct, and intellectual contribution to the work and approved it for publication.

## Funding

This work was supported by a Shenzhen Committee of Scientific and Technical Innovation grant (JCYJ20180508152244835), the Sanming Project of Medicine in Shenzhen (SZSM202011008), a Shenzhen Key Medical Discipline Construction Fund (SZXK064), and the Science & Technology Department of Sichuan Province research project (2021YFS0005 & 2021YFQ0060).

## Conflict of Interest

The authors declare that the research was conducted in the absence of any commercial or financial relationships that could be construed as a potential conflict of interest.

## Publisher’s Note

All claims expressed in this article are solely those of the authors and do not necessarily represent those of their affiliated organizations, or those of the publisher, the editors and the reviewers. Any product that may be evaluated in this article, or claim that may be made by its manufacturer, is not guaranteed or endorsed by the publisher.
