# Supplementary material for: Three Specific Potential Epitopes That Could Be Recognized by T Cells of Convalescent COVID-19 Patients Were Identified From Spike Protein
Source: Front Immunol. 2022 Jan 28;13:752622. doi: 10.3389/fimmu.2022.752622 (PMC8831549; doi:10.3389/fimmu.2022.752622)
Supplement: Supplementary file 1 [file DataSheet_1.docx]

Supplementary Material

# Supplementary Tables

**Table. S1** Peptides sequence of overlapping peptide library

| P1 | MFVFLVLLPLVSSQC | P2 | VSSQCVNLTTRTQLP |
| --- | --- | --- | --- |
| P3 | RTQLPPAYTNSFTRG | P4 | \| SFTRGVYYPDKVFRS \| \| --- \| |
| P5 | KVFRSSVLHSTQDLF | P6 | TQDLFLPFFSNVTWF |
| P7 | NVTWFHAIHVSGTNG | P8 | SGTNGTKRFDNPVLP |
| P9 | NPVLPFNDGVYFAST | P10 | YFASTEKSNIIRGWI |
| P11 | IRGWIFGTTLDSKTQ | P12 | DSKTQSLLIVNNATN |
| P13 | NNATNVVIKVCEFQF | P14 | CEFQFCNDPFLGVYY |
| P15 | LGVYYHKNNKSWMES | P16 | SWMESEFRVYSSANN |
| P17 | SSANNCTFEYVSQPF | P18 | VSQPFLMDLEGKQGN |
| P19 | GKQGNFKNLREFVFK | P20 | EFVFKNIDGYFKIYS |
| P21 | FKIYSKHTPINLVRD | P22 | NLVRDLPQGFSALEP |
| P23 | SALEPLVDLPIGINI | P24 | IGINITRFQTLLALH |
| P25 | LLALHRSYLTPGDSS | P26 | PGDSSSGWTAGAAAY |
| P27 | GAAAYYVGYLQPRTF | P28 | QPRTFLLKYNENGTI |
| P29 | ENGTITDAVDCALDP | P30 | CALDPLSETKCTLKS |
| P31 | CTLKSFTVEKGIYQT | P32 | GIYQTSNFRVQPTES |
| P33 | QPTESIVRFPNITNL | P34 | NITNLCPFGEVFNAT |
| P35 | VFNATRFASVYAWNR | P36 | YAWNRKRISNCVADY |
| P37 | CVADYSVLYNSASFS | P38 | SASFSTFKCYGVSPT |
| P39 | GVSPTKLNDLCFTNV | P40 | CFTNVYADSFVIRGD |
| P41 | VIRGDEVRQIAPGQT | P42 | APGQTGKIADYNYKL |
| P43 | YNYKLPDDFTGCVIA | P44 | GCVIAWNSNNLDSKV |
| P45 | LDSKVGGNYNYLYRL | P46 | YLYRLFRKSNLKPFE |
| P47 | LKPFERDISTEIYQA | P48 | EIYQAGSTPCNGVEG |
| P49 | NGVEGFNCYFPLQSY | P50 | PLQSYGFQPTNGVGY |
| P51 | NGVGYQPYRVVVLSF | P52 | VVLSFELLHAPATVC |
| P53 | PATVCGPKKSTNLVK | P54 | TNLVKNKCVNFNFNG |
| P55 | FNFNGLTGTGVLTES | P56 | VLTESNKKFLPFQQF |
| P57 | PFQQFGRDIADTTDA | P58 | DTTDAVRDPQTLEIL |
| P59 | TLEILDITPCSFGGV | P60 | SFGGVSVITPGTNTS |
| P61 | GTNTSNQVAVLYQDV | P62 | LYQDVNCTEVPVAIH |
| P63 | PVAIHADQLTPTWRV | P64 | PTWRVYSTGSNVFQT |
| P65 | NVFQTRAGCLIGAEH | P66 | IGAEHVNNSYECDIP |
| P67 | ECDIPIGAGICASYQ | P68 | CASYQTQTNSPRRAR |
| P69 | PRRARSVASQSIIAY | P70 | SIIAYTMSLGAENSV |
| P71 | AENSVAYSNNSIAIP | P72 | SIAIPTNFTISVTTE |
| P73 | SVTTEILPVSMTKTS | P74 | MTKTSVDCTMYICGD |
| P75 | YICGDSTECSNLLLQ | P76 | NLLLQYGSFCTQLNR |
| P77 | TQLNRALTGIAVEQD | P78 | AVEQDKNTQEVFAQV |
| P79 | VFAQVKQIYKTPPIK | P80 | TPPIKDFGGFNFSQI |
| P81 | NFSQILPDPSKPSKR | P82 | KPSKRSFIEDLLFNK |
| P83 | LLFNKVTLADAGFIK | P84 | AGFIKQYGDCLGDIA |
| P85 | LGDIAARDLICAQKF | P86 | CAQKFNGLTVLPPLL |
| P87 | LPPLLTDEMIAQYTS | P88 | AQYTSALLAGTITSG |
| P89 | TITSGWTFGAGAALQ | P90 | GAALQIPFAMQMAYR |
| P91 | QMAYRFNGIGVTQNV | P92 | VTQNVLYENQKLIAN |
| P93 | KLIANQFNSAIGKIQ | P94 | IGKIQDSLSSTASAL |
| P95 | TASALGKLQDVVNQN | P96 | VVNQNAQALNTLVKQ |
| P97 | TLVKQLSSNFGAISS | P98 | GAISSVLNDILSRLD |
| P99 | LSRLDKVEAEVQIDR | P100 | VQIDRLITGRLQSLQ |
| P101 | LQSLQTYVTQQLIRA | P102 | QLIRAAEIRASANLA |
| P103 | SANLAATKMSECVLG | P104 | ECVLGQSKRVDFCGK |
| P105 | DFCGKGYHLMSFPQS | P106 | SFPQSAPHGVVFLHV |
| P107 | VFLHVTYVPAQEKNF | P108 | QEKNFTTAPAICHDG |
| P109 | ICHDGKAHFPREGVF | P110 | REGVFVSNGTHWFVT |
| P111 | HWFVTQRNFYEPQII | P112 | EPQIITTDNTFVSGN |
| P113 | FVSGNCDVVIGIVNN | P114 | GIVNNTVYDPLQPEL |
| P115 | LQPELDSFKEELDKY | P116 | ELDKYFKNHTSPDVD |
| P117 | SPDVDLGDISGINAS | P118 | GINASVVNIQKEIDR |
| P119 | KEIDRLNEVAKNLNE | P120 | KNLNESLIDLQELGK |
| P121 | QELGKYEQYIKWPWY | P122 | KWPWYIWLGFIAGLI |
| P123 | IAGLIAIVMVTIMLC | P124 | TIMLCCMTSCCSCLK |
| P125 | CSCLKGCCSCGSCCK | P126 | GSCCKFDEDDSEPVL |
| P127 | SEPVLKGVKLHYT |  |  |

**Table. S2** Checkerboard list of peptides

| Group | C1 | C2 | C3 | C4 | C5 | C6 | C7 | C8 | C9 | C10 | C11 | C12 |
| --- | --- | --- | --- | --- | --- | --- | --- | --- | --- | --- | --- | --- |
| R1 | P1 | P2 | P3 | P4 | P5 | P6 | P7 | P8 | P9 | P10 | P11 | P12 |
| R2 | P13 | P14 | P15 | P16 | P17 | P18 | P19 | P20 | P21 | P22 | P23 | P24 |
| R3 | P25 | P26 | P27 | P28 | P29 | P30 | P31 | P32 | P33 | P34 | P35 | P36 |
| R4 | P37 | P38 | P39 | P40 | P41 | P42 | P43 | P44 | P45 | P46 | P47 | P48 |
| R5 | P49 | P50 | P51 | P52 | P53 | P54 | P55 | P56 | P57 | P58 | P59 | P60 |
| R6 | P61 | P62 | P63 | P64 | P65 | P66 | P67 | P68 | P69 | P70 | P71 | P72 |
| R7 | P73 | P74 | P75 | P76 | P77 | P78 | P79 | P80 | P81 | P82 | P83 | P84 |
| R8 | P85 | P86 | P87 | P88 | P89 | P90 | P91 | P92 | P93 | P94 | P95 | P96 |
| R9 | P97 | P98 | P99 | P100 | P101 | P102 | P103 | P104 | P105 | P106 | P107 | P108 |
| R10 | P109 | P110 | P111 | P112 | P113 | P114 | P115 | P116 | P117 | P118 | P119 | P120 |
| R11 | P121 | P122 | P123 | P124 | P125 | P126 | P127 |  |  |  |  |  |

**Table. S3** Basic information of convalescent COVID-19 patients

| characteristics | | number | percent% |
| --- | --- | --- | --- |
| gender | male | 31 | 43.7 |
|  | female | 40 | 56.3 |
| Age groups | 0-19 | 11 | 15.5 |
|  | 20-59 | 40 | 56.3 |
|  | 60-89 | 8 | 11.3 |
|  | NA | 12 | 16.9 |
| disease severity | asymptomatic | 7 | 9.9 |
|  | mild | 9 | 12.7 |
|  | moderate | 35 | 49.3 |
|  | sever | 2 | 2.8 |
|  | critical | 1 | 1.4 |
|  | NA | 17 | 23.9 |
| S/CO value of RBD total antibody (COI) | 600-1000 | 5 | 7.1 |
|  | 300-600 | 14 | 19.7 |
|  | 0-300 | 39 | 54.9 |
|  | NA | 13 | 18.3 |

**Table. S4** conservation analysis of seven peptides among coronavirus S protein

| peptide | sequence | coronavirus | sequence ^g^ | conservation /% |
| --- | --- | --- | --- | --- |
| P14 | CEFQFCNDPFLGVYY | 229E^a^ | YSITPCNPPDQLVVY | 33.33 |
|  |  | NL63^b^ | PTHWFCANQSTSVYS | 26.67 |
|  |  |  | NGFPFNNWFLLTNGS |  |
|  |  |  | YSANFLDDNVLPETY |  |
|  |  |  | FIVTPCNQPDQVAVY |  |
|  |  |  | SNFGICADGSLIPVR |  |
|  |  |  | NAFSLANVTSFGDYN |  |
|  |  |  | VKPNFDLTPFNLTYL |  |
|  |  |  | VELQGLIDQINSTYV |  |
|  |  | OC43^c^ | CTSDNINDKDTGPPP | 26.67 |
|  |  |  | STDTVDVTNGLGTYY |  |
|  |  |  | DRVYLNTTLFLNGYY |  |
|  |  |  | TLFLNGYYPTSGSTY |  |
|  |  |  | FLSDFINGIFAKVKN |  |
|  |  |  | EYPQTICHPNLGNHR |  |
|  |  |  | FLFNVYLGMALSHYY |  |
|  |  |  | KCKTQSIAPPTGVYE |  |
|  |  |  | DLQKANTDIILGVCV |  |
|  |  |  | TQLQVANSLMNGVTL |  |
|  |  |  | FKNQTSVAPDLSLDY |  |
|  |  | HKU1^d^ | SEYVVDVSYGLGTYY | 33.33 |
|  |  |  | ASCLCSTDAFLGWSY |  |
|  |  |  | LNLHTINATFLDLYY |  |
|  |  | SARS^e^ | CNFELCDNPFFAVSK | 40 |
|  |  | MERS^f^ | LEPRSGNHCPAGNSY | 26.67 |
|  |  |  | EYFNLRNCTFMYTYN |  |
|  |  |  | NMFQFATLPVYDTIK |  |
|  |  |  | HCSYESFDVESGVYS |  |
|  |  |  | GRGVFQNCTAVGVRQ |  |
|  |  |  | QRFVYDAYQNLVGYY |  |
|  |  |  | TGFTTTNEAFQKVQD |  |
|  |  |  | IDFQDELDEFFKNVS |  |
| P24 | IGINITRFQTLLALH | 229E^a^ | TGVNDAITQTSQALQ | 40 |
|  |  | NL63^b^ | LGITISGETVRLHLY | 33.33 |
|  |  |  | SSVNDAITQTAEAIH |  |
|  |  |  | FSIVNSAPDGLLFLH |  |
|  |  |  | WLIISVVFVVLLSLL |  |
|  |  | OC43^c^ | WVTPLTSRQYLLAFN | 33.33 |
|  |  | HKU1^d^ | MLLIIFILPTTLAVI | 26.67 |
|  |  |  | NGIFSRVKNTKLYVN |  |
|  |  |  | DSGMPTTFLFSLYLG |  |
|  |  |  | FSNCNFNLSTLLRLV |  |
|  |  |  | IGTNYRSCESTTVLD |  |
|  |  |  | NGINSGTTCSNDLLQ |  |
|  |  |  | INSGTTCSNDLLQPN |  |
|  |  |  | DNINFKSLVGCLGPH |  |
|  |  |  | LSIQNGFSATNSALA |  |
|  |  |  | VQIDRLINGRLTALN |  |
|  |  |  | IDRLINGRLTALNAY |  |
|  |  |  | SDISLVKFGAALAME |  |
|  |  |  | WFKNQTSIAPNLTLN |  |
|  |  |  | TLNLHTINATFLDLY |  |
|  |  |  | ISFSFIIFLVLLFFI |  |
|  |  | SARS^e^ | LGINITNFRAILTAF | 46.67 |
|  |  | MERS^f^ | YGGNMFQFATLPVYD | 33.33 |
|  |  |  | IGDIIQRLDVLEQDA |  |
|  |  |  | FGSLTQINTTLLDLT |  |
| P38 | SASFSTFKCYGVSPT | 229E^a^ | SASINTGNCPFSFGK | 33.33 |
|  |  |  | YASNGTYNCTDAVLT |  |
|  |  |  | LANVSSFGDYNLSSV |  |
|  |  | NL63^b^ | SATNIQNLLYCDSPF | 33.33 |
|  |  |  | SPGDSSWHIYLKSGT |  |
|  |  | OC43^c^ | SDFMSEIKCKTQSIA | 33.33 |
|  |  | HKU1^d^ | SYRFVTFEPFNVSFV | 40 |
|  |  |  | SYKPISFKTVLVSPG |  |
|  |  | SARS^e^ | STFFSTFKCYGVSAT | 80 |
|  |  | MERS^f^ | NASLNSFKEYFNLRN | 33.33 |
|  |  |  | LFSVNDFTCSQISPA |  |
| P48 | EIYQAGSTPCNGVEG | 229E^a^ | EYLQITSTPIVVDCS | 33.33 |
|  |  | NL63^b^ | FLLTNGSTLVDGVSR | 33.33 |
|  |  |  | EYLQITSTPIVVDCA |  |
|  |  | OC43^c^ | SFIQADSFTCNNIDA | 33.33 |
|  |  | HKU1^d^ | NSYTIVVQPHNGVLE | 33.33 |
|  |  | SARS^e^ | GIYFAATEKSNVVRG | 40 |
|  |  | MERS^f^ | IIYPQGRTYSNITIT | 33.33 |
|  |  |  | TITYQGLFPYQGDHG |  |
|  |  |  | STYGPLQTPVGCVLG |  |
|  |  |  | SSYFKLSIPTNFSFG |  |
| P74 | MTKTSVDCTMYICGD | 229E^a^ | MNVTLNKCTKYNIYD | 40 |
|  |  |  | STPIVVDCSTYVCNG |  |
|  |  | NL63^b^ | STPIVVDCATYVCNG | 40 |
|  |  | OC43^c^ | SPKVTIDCAAFVCGD | 40 |
|  |  | HKU1^d^ | NTKLYVNKTLYSEFS | 33.33 |
|  |  |  | NFKSLVGCLGPHCGS |  |
|  |  | SARS^e^ | MAKTSVDCNMYICGD | 86.67 |
|  |  | MERS^f^ | IQKVTVDCKQYVCNG | 40 |
| P77 | TQLNRALTGIAVEQD | 229E^a^ | LLVAYALLHIAGCQT | 33.33 |
|  |  |  | TQTSQALQTVATALN |  |
|  |  |  | KWLNRVETYIKWPWW |  |
|  |  | NL63^b^ | TEQLGAPLGITISGE | 26.67 |
|  |  |  | TTHNGRVVNYTVCDD |  |
|  |  |  | HQVNISLNGNTSVCV |  |
|  |  |  | EYLQITSTPIVVDCA |  |
|  |  |  | PQRNIRSSRIAGRSA |  |
|  |  |  | LALQARLNYVALQTD |  |
|  |  |  | ARLNYVALQTDVLQE |  |
|  |  |  | SSVNDAITQTAEAIH |  |
|  |  |  | QQVDRLITGRLAALN |  |
|  |  |  | YKNVKAWSGICVDGI |  |
|  |  |  | TYLNLSSELKQLEAK |  |
|  |  |  | KLLNRFENYIKWPWW |  |
|  |  | OC43^c^ | ILLISLPTAFAVIGD | 33.33 |
|  |  |  | TYRNMALKGSVLLSR |  |
|  |  |  | TPLTSRQYLLAFNQD |  |
|  |  |  | TDLQKANTDIILGVC |  |
|  |  |  | NAFNNALYAIQEGFD |  |
|  |  | HKU1^d^ | CQLYYSLPAINVTIN | 33.33 |
|  |  |  | TQLHVADTLMQGVTL |  |
|  |  |  | TAFNNALLSIQNGFS |  |
|  |  | SARS^e^ | TQLNRALSGIAAEQD | 86.67 |
|  |  | MERS^f^ | SKINQALHGANLRQD | 40 |
| P84 | AGFIKQYGDCLGDIA | 229E^a^ | TGFVYFNGTGRGDCQ | 33.33 |
|  |  | NL63^b^ | LLFTEQLGAPLGITI | 33.33 |
|  |  | OC43^c^ | DGIIFNAEDCMSDFM | 33.33 |
|  |  |  | IDAAKIYGMCFSSIT |  |
|  |  |  | VGFVEAYNNCTGGAE |  |
|  |  | HKU1^d^ | FDESKIYGSCFKSII | 33.33 |
|  |  |  | VGFVEAYNNCTGGSE |  |
|  |  | SARS^e^ | AGFMKQYGECLGDIN | 80 |
|  |  | MERS^f^ | IGDIIQRLDVLEQDA | 40 |

a: spike protein of human coronavirus 229E (GenBank: BAL45639.1).

b: spike protein of human coronavirus NL63 (GenBank: AFD98834.1).

c: spike protein of human coronavirus OC43 (GenBank: AAA03055.1).

d: spike protein of human coronavirus HKU1 (GenBank: BBA20983.1).

e: spike protein of SARS coronavirus (GenBank: ABA02260.1).

f: spike protein of MERS coronavirus (NCBI Reference Sequence: YP_009047204.1)

## g: The red part represents different amino acids.

## Supplementary Figures


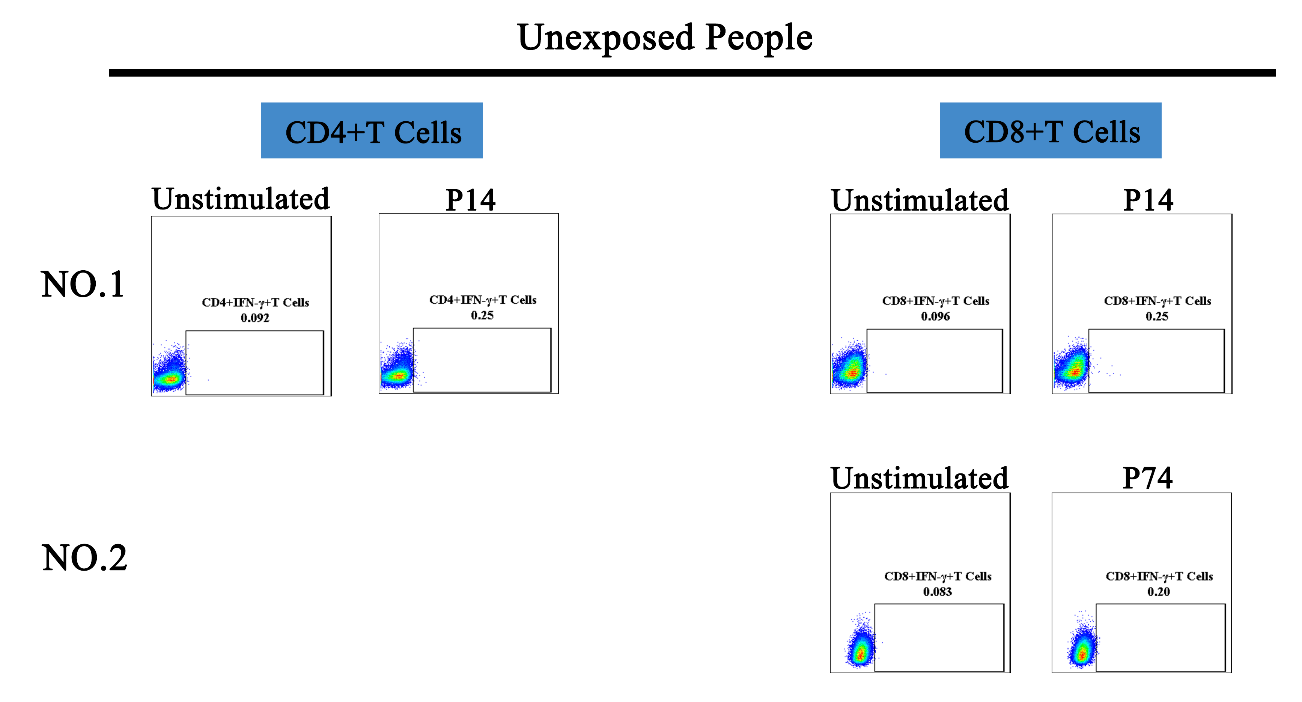


**Supplementary Figure 1.** Cross reaction of peptides in healthy people. The seven peptides (P14, P24, P38, P48, P74, P77 and P84) screened in this study were tested in 14 PBMC samples of healthy people. The results only showed T cell positive response. CD4^+^IFN-γ^+^T and CD8^+^IFN-γ^+^T cell positive response induced by P14 were detected in one sample (NO. 1), and CD8^+^IFN-γ^+^T cell positive response induced by P14 was detected in another sample. The remaining samples did not show T cell positive response to peptides.


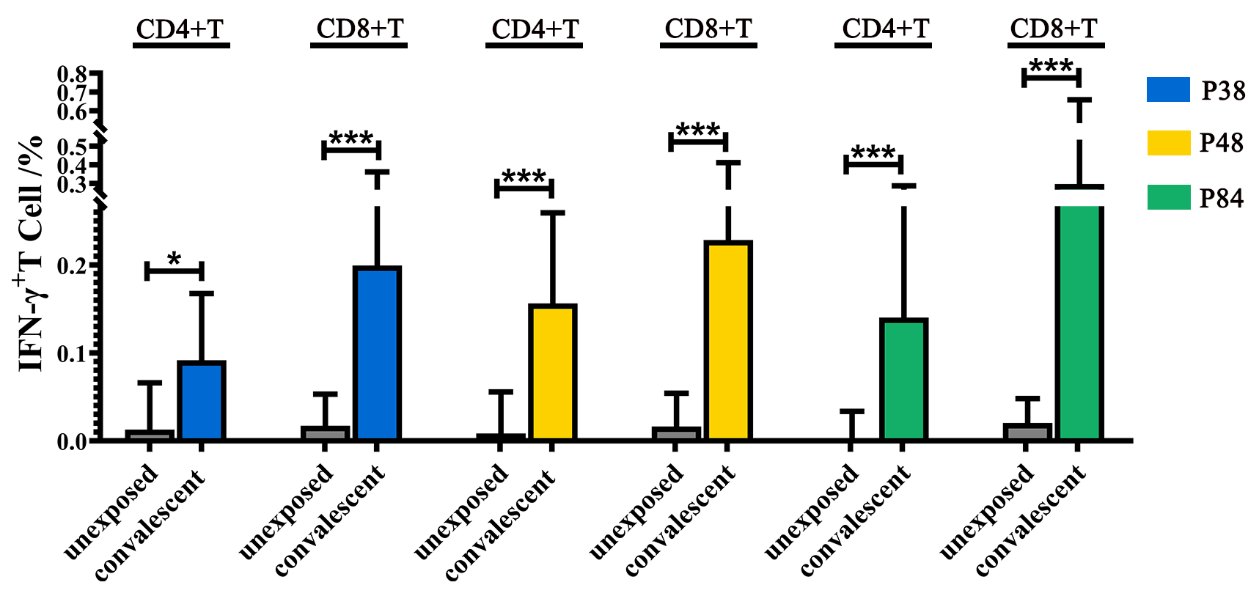


**Supplementary Figure 2.** T cell response of unexposed population and convalescent COVID-19 patients to the three potential epitopes. The data showed the proportion value of CD4^+^ IFN-γ^+^ T cells and CD8^+^ IFN-γ^+^ T cells induced by the potential epitopes (P38, P48 and P84) in the PBMCs of unexposed people (n=14, grey bars), and the positive proportion value of CD4^+^ IFN-γ^+^ T cells and CD8^+^ IFN-γ^+^ T cells detected in the PBMCs of convalescent COVID-19 patients (colored bars). *: *P*<0.05, **: *P*<0.01, ***: *P*<0.001.


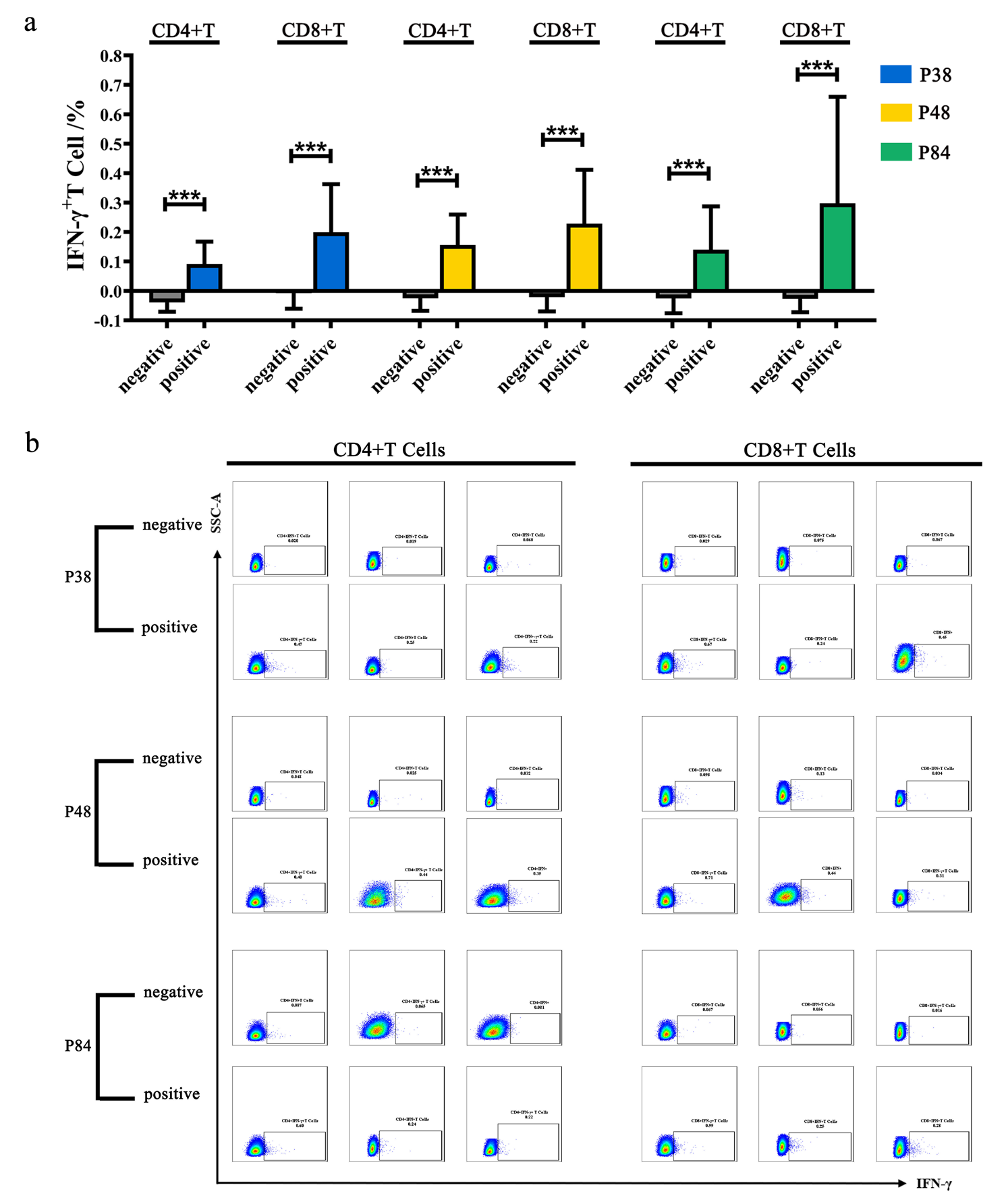


**Supplementary Figure 3.** T cell response of convalescent COVID-19 patients to the three potential epitopes. **a** The data showed the IFN-γ^+^ T cells proportion value of negative responses (grey bars) and positive responses (colored bars) induced by the potential epitopes (P38, P48 and P84) in the PBMCs of convalescent COVID-19 patients. *: *P*<0.05, **: *P*<0.01, ***: *P*<0.001. **b** Part of the original flow scatter plots of IFN-γ^+^ CD4^+^T and IFN-γ^+^ CD8^+^T cells.
